# The prevalence of uterine anomalies in infertile patients with polycystic ovary syndrome: A retrospective study in a tertiary center in Southeastern Turkey

**DOI:** 10.4274/tjod.galenos.2019.62589

**Published:** 2020-02-28

**Authors:** Serhat Ege, Nurullah Peker, Muhammed Hanifi Bademkıran

**Affiliations:** 1 Diyarbakır University of Health Sciences, Gazi Yaşargil Training and Research Hospital, Clinic of Obstetrics and Gynecology, Diyarbakır, Turkey; 2 Dicle University Faculty of Medicine, Department of Obstetrics and Gynecology, Diyarbakır, Turkey

**Keywords:** Uterine anomaly, polycystic ovary syndrome, infertility

## Abstract

**Objective:**

To evaluate the prevalence of uterine anomalies in infertile patients with polycystic ovary syndrome (PCOS) admitted to our tertiary hospital in Southeastern Turkey.

**Materials and Methods:**

The files of 3033 patients with infertility who presented to the infertility polyclinics were retrospectively analyzed, and uterine anomalies were detected in 131 patients. Seven hundred ten of these patients were evaluated as having PCOS, 55 of whom had uterine anomalies. Patients with PCOS were also divided into two subgroups as those with primary and secondary infertility.

**Results:**

Of the 3033 patients with infertility who were evaluated, 57 (8%) of 710 infertile patients with PCOS, and 74 (3%) of 2323 non-PCOS patients with infertility had uterine anomalies. A statistically significant difference was found between the two groups (p<0.001), and no significant difference was found between the primary and secondary infertile PCOS subgroups (p=0.3). Septate uteri and arcuate uteri had a high prevalence in the PCOS group, and no t-shaped or hypoplastic uteruses were observed in this group.

**Conclusion:**

To or knowledge, this is the first study in our region to examine the relation between PCOS and Müllerian anomalies. We demonstrated uterine anomalies and their prevalence in patients with infertility. A more careful examination is required in order to determine the incidence of uterine anomalies in patients with PCOS.

**PRECIS:** This is the first study in our region that we know of that offers an examination of the relation between polycystic ovary syndrome and Müllerian anomalies.

## Introduction

Polycystic ovary syndrome (PCOS) is a heterogeneous, multifactorial disease affecting 10% of the female population of reproductive age. Hyperandrogenism, ovulatory dysfunction, and polycystic ovary images are among the main features of PCOS^(1,2)^. Congenital uterine anomalies occur as a result of a defect in the Müllerian canals. Genetic, sporadic or multifactorial factors are thought to play a role in the formation of Müllerian duct anomalies. Uterine anomalies cause decrease pregnancy rates and increase the risk of miscarriage and preterm birth^(3,4,5)^. The high rates of PCOS and uterine anomalies in patients with infertility suggest that there may be a relationship between them. This study aimed to evaluate the prevalence of uterine anomalies in infertile patients with PCOS in a tertiary center in Southeast Turkey.

## Materials and Methods

The files of 3033 infertile patients who presented to the infertility polyclinics of Gazi Yaşargil Training and Research Hospital in the Southeast part of Turkey between February 2017 and May 2019 were retrospectively analyzed, and uterine anomalies were detected in 131 patients. Of all the infertile patients, 710 were evaluated as having PCOS, and 57 had uterine anomalies. Patients with PCOS were divided into two subgroups, as those with primary or secondary infertility.

The patients were evaluated according to the Rotterdam criteria: 1: ultrasound examination; 2: clinical and biochemical evidence of hyperandrogenism; and 3: oligoovulation/anovulation^(6)^.

All patients with myoma, ovary cysts, tubular blockage, and male-factor infertility were excluded. The patients were first evaluated using transvaginal ultrasonography. Standard steps hysterosalpingography, laparoscopy, hysteroscopy, and magnetic resonance imaging were performed to confirm the diagnosis of uterine anomalies. The American Fertility Society classification was used to diagnose uterine anomalies^(7)^. The study was approved by the Local Ethics Committee of  University of Health Sciences Gazi Yaşargil Training and Research Hospital (approval number: 318).

### Statistical Analysis

The data of nominal variables are summarized in the form of frequency or percentages. Comparative data were compared using the chi-square test. Any differences were considered significant for p values smaller than 0.05. All statistical analyses were performed using R-software v.3.5.1 (R statistics software, Institute for Statistics and Mathematics, Vienna, Austria).

## Results

Of the 3033 patients with infertility who were evaluated, 57 (8%) of the 710 infertile patients with PCOS and 74 (3%) of the 2323 non-PCOS patients with infertility had uterine anomalies. Septate uteri and arcuate uteri had a high prevalence in the PCOS group, and no t-shaped or hypoplastic uteruses were observed in this group (Table 1). A statistically significant difference was found between the two groups (p<0.001), and no significant difference was found between the primary and secondary infertile PCOS subgroups (p=0.3) (Table 2).

## Discussion

It is necessary to diagnose uterine anomalies because of their different structural features^(8)^. The incidence of uterine anomalies was reported as 6.7% in fertile patients, 7.3% in infertile patients, and 16.7% in recurrent abortions^(9)^. In the present study, the prevalence of uterine anomalies in infertile patients with PCOS admitted to our hospital was evaluated retrospectively, and a significant relationship was observed between PCOS and uterine anomalies. The reproductive system, except the ovaries, consists of müllerian channels. The uterus, which is composed of mullerian canals, is initially separated by a septum, then fusion occurs when the intervening septum disappears^(5)^. It is thought that the uterine septum is regressed by mediation of the *Bcl-2* gene^(10)^. Defects in the formation, convergence or regression of the Müllerian ducts can cause different anomalies. Several studies have shown significantly higher anti-Müllerian hormone (AMH) levels in patients with PCOS. This could be linked to coexisting uterine anomalies because AMH definitely plays a role during early life in the degeneration of Müllerian ducts^(11,12,13)^. In the present study, a significant relationship was noted between PCOS and uterine anomalies. Developmental defects could be a possible cause for both PCOS and uterine anomalies. Hormonal changes such as AMH may play a role in the etiopathogenesis of both conditions. MacDougall and Ultrasonographer^(14) ^noted that in 1512 women with PCOS, two cases of Müllerian anomalies were reported, and their prevalence in patients with PCOS and the general population was not different. Similarly, Acién^(15)^ found no such relationship of polycystic ovarian disease in women with uterine malformations. In contrast, Appelman et al.^(16)^ found a significant relationship between PCOS and uterine Müllerian anomalies. In a retrospective study, it was reported by Ugur et al.^(17)^ that PCOS and Müllerian anomalies occurred as a result of a common. Finally, Saleh and Shawky Moiety^(18)^ found a relationship between PCOS and uterine anomalies in a prospective study of patients with infertility. In addition, uterine anomaly classification, arcuate uteri was the most common anomaly in PCOS patients, followed by septum uteri. Similarly, in this study, septate uteri and arcuate uteri had a high prevalence level in the PCOS group. Christiansen and Detti^(19)^ showed that surgical correction was warranted for Müllerian anomalies that caused pregnancy loss, such as septate and subseptate (i.e., arcuate) uteri. The minimum subseptation length that indicates a surgical incision is still debated; however, authors advocated a 5.9 mm cut-off and proposed that it be adopted, especially when a history of pregnancy loss is present or when fertility treatments are planned.

The limitation of the study is that the prevalence of PCOS did not reflect the general population because the patient sample mainly comprised women with infertility. This may be due to the fact that patients without PCOS with Müllerian anomalies do not present as infertile. It should also be noted that it is not easy to perform an epidemiologic study on the prevalence of Müllerian anomalies because they are not common in the general population, and most go undiagnosed because they are asymptomatic. To our knowledge, this is the first study in our region to examine the relation between PCOS and Müllerian anomalies. We demonstrated uterine anomalies and their prevalence in patients with infertility. A more careful examination is required in order to determine the incidence of uterine anomalies in patients with PCOS.

## Figures and Tables

**Table 1:**
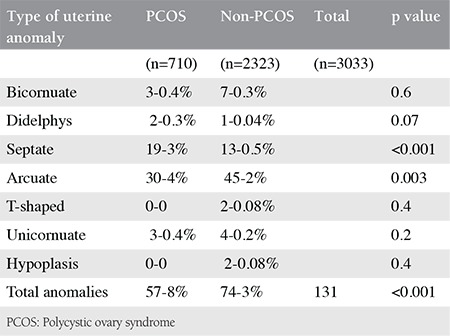
Distribution of uterine anomalies

**Table 2:**
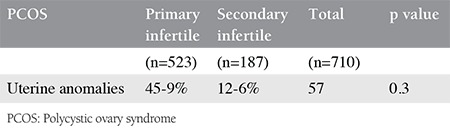
Distribution of cases with polycystic ovary syndrome and uterine anomalies
